# Appraising the role of circulating concentrations of micronutrients in attention deficit hyperactivity disorder: a Mendelian randomization study

**DOI:** 10.1038/s41598-023-49283-y

**Published:** 2023-12-09

**Authors:** Xiaohui Sui, Tingting Liu, Zhiyun Zou, Baoqing Zhang

**Affiliations:** 1 Shandong University of Traditional Chinese Medicine, Jinan, 250014 Shandong China; 2 Affiliated Hospital of Shandong University of Traditional Chinese Medicine, Jinan, 250011 Shandong China

**Keywords:** Paediatric neurological disorders, Paediatric research

## Abstract

Previous observational researches have discovered a connection between circulating concentrations of micronutrients and attention deficit hyperactivity disorder (ADHD). However, the results may be influenced by confounding factors and reverse causation. This study aims to explore the causal relationship between circulating concentrations of micronutrients and ADHD using Mendelian randomization (MR). In a two-sample MR context, we used summary data from the major European genome-wide association studies (GWAS) for these illnesses to assess the genetically anticipated effects of circulating concentrations of micronutrients on ADHD risk. In order to achieve this, we took single nucleotide polymorphisms (SNPs) from the GWAS that were highly related with concentrations of nine micronutrients. The corresponding data for ADHD were extracted from the Psychiatric Genomics Consortium. Inverse-variance weighted (IVW) method was used as the main MR analysis, and the reliability of the study’s conclusions was assessed using sensitivity analyses. Our MR analyses showed that the copper level may be associated with a reduced risk of ADHD. However, the significance of the research results is weak. There were no clear relationships between other micronutrients and ADHD. Our sensitivity studies confirmed the findings of the primary IVW MR analyses. According to this study, there may be some association between copper level and ADHD, but the significance of the research results is weak, and it is recommended that copper level should be used as a long-term monitoring indicator for further research. The results provide a new idea for the further study of ADHD, and provide guidance for the prevention and treatment of ADHD.

## Introduction

In children and adolescents, attention deficit hyperactivity disorder (ADHD) is a prevalent mental disorders. The main clinical manifestations are age-inappropriate obliviousness, impulsivity, hyperactivity and other behaviors^1,2^. According to the study, one of the most prevalent childhood problems is ADHD, which affects 7.2% of children^3^. As a complex disease caused by genetic, social and environmental factors, ADHD symptoms can last for several years, and have extensive and lasting negative effects on children's study, work, life and other aspects^2,4,5^. At the same time, the potential link between micronutrients and ADHD has attracted extensive attention from researchers, which can offer fresh suggestions for ADHD treatment.

Micronutrients include vitamins and minerals needed by the human body, which have a key part in the growth and development of the human body and the prevention and treatment of diseases. Previous research has found the correlation between several serum micronutrients (magnesium, iron, copper, zinc, selenium, vitamin A, vitamin B12, vitamin D and folate) and psychiatric disorders such as ADHD^6–10^. Kids with ADHD reported significantly lower levels of iron, magnesium, zinc, and vitamin D compared with normal healthy children, suggesting a link between micronutrients and mental disorders^11^, it is unknown if these relationships are causative. Mendelian randomization (MR), which uses genetic variation as an instrumental variable, can be used to determine whether there is a probable causal association between exposures and disease^12–14^.

MR is an important tool in epidemiological studies, can effectively use the Genome—wide Association Study (GWAS) data as a result, genetic variation as the instrumental variables (IVs), to investigate the relationship between an interesting exposure and an outcome^14,15^. As the genotype is assigned at random during conception, genetic variants are unaffected by potential confounding factors, so it can overcome the limitation of traditional observational research^16,17^.

We investigated the probable causal connection between nine micronutrients (zinc, iron, copper, magnesium, selenium, vitamin A, vitamin B12, vitaminD and folate) and ADHD in the present study, using a two-sample Mendelian randomization study. Our results will provide new directions for the mechanisms and treatment of ADHD.

## Materials and methods

### Study design

The two-sample MR approach was used to examine the potential causal relationship between the concentration of circulating micronutrients and ADHD. The reliability of the results is tested by sensitivity analysis. To ensure the reliability of the results, MR is based on three critical assumptions^15,18^: (1) IVs and the concentration of circulating micronutrients are strongly associated, (2) There are no confounders influencing both exposure and outcome that are related to IVs, (3) IVs only affect ADHD through circulating micronutrient concentration. This study followed the recommendations of the STROBE-MR^19^. Figure S1 depicts our study design.

### ADHD population

Genotype data on ADHD were extracted from the Psychiatric Genomics Consortium (PGC)^20^. The PGC aimed to facilitate quick progress in revealing the genetic basis of psychiatric diseases by unifying most of the field’s genetics research^21^. There were 20,183 individuals diagnosed with ADHD and 35,191 controls from 12 cohorts. Only individuals of white European descent were included in our research to reduce any confounding caused by ancestry (19,099 cases and 34,194 controls).

### Micronutrients GWAS sources

Published GWAS on micronutrients, were searched by using GWAS Catalog^22^, IEU OpenGWAS (https://gwas.mrcieu.ac.uk/), and Pubmed. The initial inventory contains 15 nutrients: iodine, lead, zinc, magnesium, copper, arsenic, manganese, folate, selenium, iron, and vitamins A, vitamins B1, vitamins B2, vitamins B12, and vitamins D^6–10,23,24^. Due to the lack of GWAS, Vitamins B1, B2, iodine, arsenic, manganese and lead were eliminated. In total, nine micronutrients with appropriate genetic instruments were examined in this study: magnesium, iron, copper, zinc, selenium, vitamin A, B12, D and folate^25–30^.The comprehensive details for the GWAS datasets used in this research was presented in Table 1.

**Table 1:** Detailed information of the GWAS datasets used in the present study.

Exposure	Study or consortium	PMID or GWAS ID	Sample size	SNP	Ancestry
Magnesium	Meyer TE et al.^25^	20,700,443	23,829	2,585,820	European
Iron	GIS	ieu-a-1049	23,986	2,096,457	European
Copper	Evans DM et al.^26^	23,720,494	2,603	2,543,646	European
Zinc	Evans DM et al.^26^	23,720,494	2,603	2,543,610	European
Selenium	Cornelis, M.C.et al.^27^	25,343,990	4,162	2,642,571	European
Folate	MRC-IEU^28^	ukb-b-11349	64,979	9,851,867	European
Vitamin A	Neale Lab	ukb-a-458	335,591	10,894,596	European
Vitamin B12	Fanidi A et al.^29^	30,499,135	417,580	6,476,552	European
Vitamin D	Revez JA et al.^30^	32,242,144	496,946	6,896,093	European

### Selection of genetic instrumental variables

At the genome-wide significance level (P < 5 × 10^−8^), all SNPs connected to a relevant exposure were retrieved. Since some phenotypes have fewer SNPS (less than 3), we expand the P-value to 5 × 10^−6^, or even 5 × 10^−4^, as in copper. Independent SNPs were chosen using conventional clumping factors (clumping window of 10,000 kb, LD r^2^ cutoff 0.001)^31^. These SNPs are not in LD. Phenoscanner database (http://www.phenoscanner.medschl.cam.ac.uk/) searches were done to make sure the contained SNPs weren’t connected to any known confounding factors. The strength of association between IVs and exposure factors was evaluated by calculating F statistics. The following formula was used to determine the F statistic: F = R^2^ × (N − 2)/(1 − R^2^)^32^. Only SNPs with an F statistics > 10 were used in future analyses to reduce weak instrument bias. Meanwhile, ambiguous and palindromic SNPs were removed in the harmonizing process. Finally, this research identified 279 SNPs associated with 9 circulating micronutrient concentrations as IVs, including 6 SNPs for magnesium, 129 SNPs for copper, 13 SNPs for iron, 7 SNPs for zinc, 11 SNPs for selenium, 8 SNPs for folate, 7 SNPs for vitamin A, 7 SNPs for vitamin B12, and 94 SNPs for vitamin D. The comprehensive details for genetic IVs used in this research was presented in Supplementary Table S1.

### Statistical analysis

In this study, five methods, such as inverse-variance weighted (IVW), MR-Egger, weighted median, modal-based simple estimation, and modal-based weighted estimation, were used to conduct Mendelian randomization analysis of two samples, and the odds ratio (OR) value and 95% confidence interval (CI) were used to evaluate the potential causal relationship between micro-nutrients and ADHD^33,34^. The key method for determining the impact of exposure on outcomes is the IVW method^35^. The Wald ratio approach is used to determine the impact of a single IV on ADHD when only one SNP is provided^36^. Different MR methods (such as MR-Egger and weighted median) were used for sensitivity analysis. To assess the degree of heterogeneity, we used Cochrane’s Q heterogeneity test, with p < 0.05 indicating a high rate of heterogeneity. When there is high heterogeneity (P < 0.05), the multiplicative random effect IVW method is used^37^. By examining whether the intercept of the connection between exposure and outcome differs from zero, the MR-Egger method can identify potential pleiotropy^36^. After adjusting for pleiotropy, the method then provides a more conservative estimate of causal effects^38^. To reduce the bias caused by horizontal pleiotropy, this study used MR-PRESSO^39^ to detect broad horizontal pleiotropy in all results. Furthermore, using leave-one-out sensitivity analysis, it was determined whether the results were influenced by individual influential SNPs. R software (version 4.1.2) was used for this study's total data analysis. The R packages used include the TwoSampleMR and MR-PRESSO packages.

## Results

### Mendelian randomization

**Figure 1:**
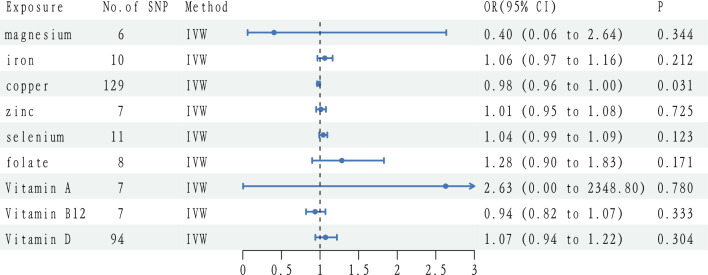
A forest plot showing associations between genetically determined levels of micronutrients and ADHD based on the wald ratio method or IVW MR analysis. *SNPs* single nucleotide polymorphism, *IVW* inverse-variance weighted, *OR* odds ratio, *CI* confidence interval.

**Figure 2:**
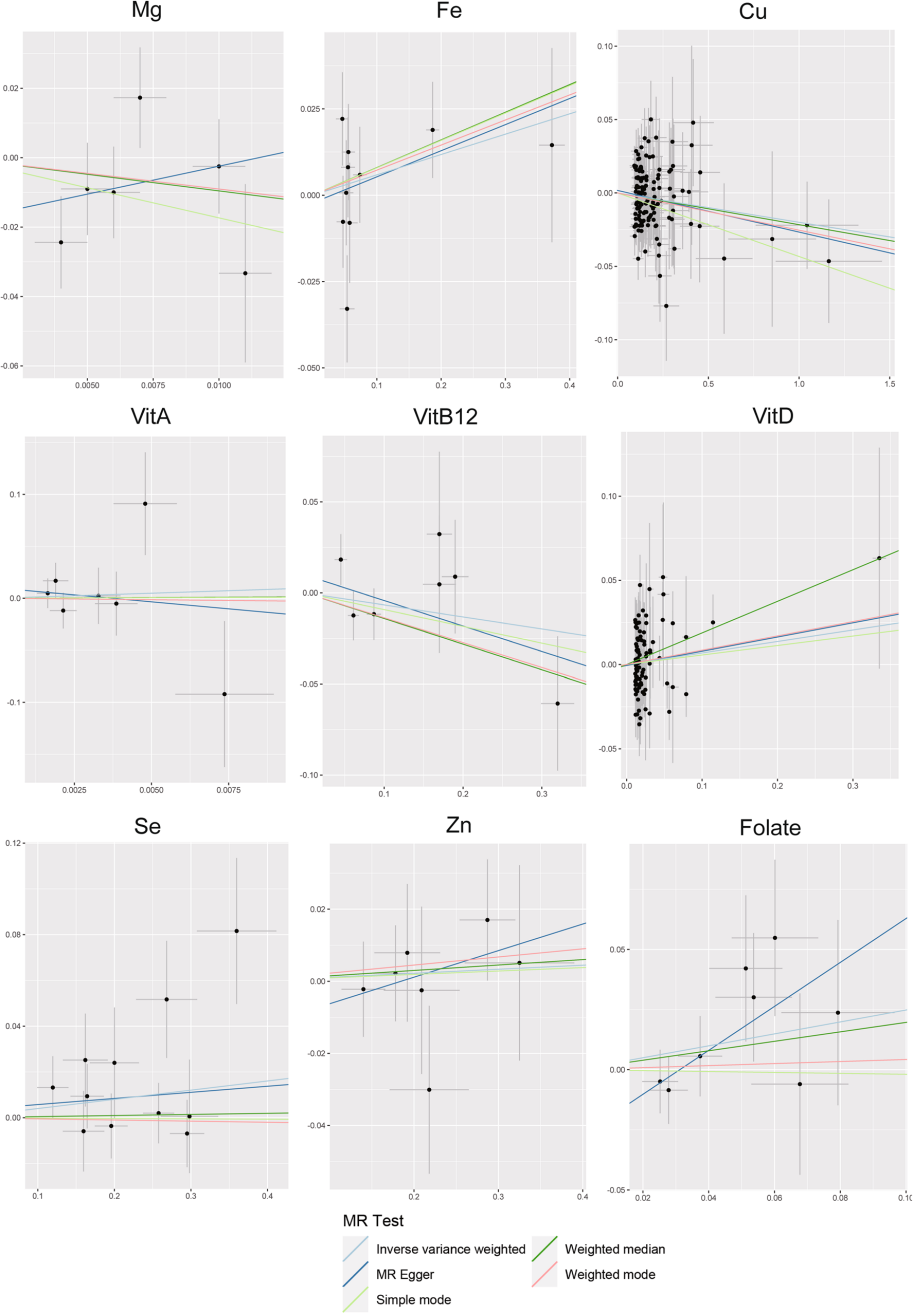
Scatter plots showing the MR effect of each exposure on ADHD. Each line represents a different MR method. *Mg* magnesium, *Fe* iron, *Cu* copper, *VitB12* vitaminB12, *VitD* vitaminD, *Se* selenium, *Zn* zinc, *MR* Mendelian randomization.

F statistics did not suggest the existence of weak instrumental bias (F > 10). Figure 1 and Table 2 demonstrate the results of evaluating the effect of circulating micronutrient concentrations on the risk of ADHD using the wald ratio method or IVW method. The genetic prediction results showed that a possible link between copper level and ADHD was found in IVW analysis (OR: 0.980, 95% CI 0.963–0.998, p = 0.031), but the findings were not significant (Fig. 1, Table 2). Other remaining blood micronutrients’ hereditary propensity for high circulating levels (magnesium (OR: 0.404, 95% CI 0.062–2.636, p = 0.344), iron (OR: 1.061, 95% CI 0.967–1.163, p = 0.212), zinc (OR: 1.011, 95% CI 0.950–1.076, p = 0.725), selenium (OR: 1.041, 95% CI 0.989–1.095, p = 0.123), vitamin A (OR: 2.628, 95% CI 0.003–2348.8, p = 0.780), B12 (OR: 0.936, 95% CI 0.819–1.070, p = 0.333), D (OR: 1.071, 95% CI 0.939–1.219, p = 0.304) and folate (OR: 1.282, 95% CI 0.898–1.829, p = 0.171)) did not associations with ADHD. This is consistent with results from the other MR methods including MR Egger and weighted median (Table 2, Fig. 2).

**Table 2:** IVW method and sensitivity analyses using WMA and MR-Egger method for the Mendelian randomization analyses of micro-nutrients and ADHD.

Exposure	N SNPs*	Method	OR	95% CI	P val
Magnesium	6	IVW	0.404	0.062–2.636	0.344
		WMA	0.384	0.045–3.290	0.383
		MR-Egger	5.016	0.014–18.33	0.621
Iron	10	IVW	1.061	0.967–1.163	0.212
		WMA	1.083	0.973–1.206	0.143
		MR-Egger	1.079	0.926–1.257	0.361
Copper	129	IVW	0.980	0.963–0.998	0.031
		WMA	0.979	0.948–1.011	0.189
		MR-Egger	0.972	0.938–1.007	0.123
Zinc	7	IVW	1.011	0.950–1.076	0.725
		WMA	1.015	0.936–1.101	0.716
		MR-Egger	1.076	0.855–1.355	0.558
Selenium	11	IVW	1.041	0.989–1.095	0.123
		WMA	1.005	0.942–1.072	0.885
		MR-Egger	1.027	0.856–1.232	0.780
Folate	8	IVW	1.282	0.898–1.829	0.171
		WMA	1.214	0.759–1.951	0.414
		MR-Egger	2.493	0.960–6.476	0.110
Vitamin A	7	IVW	2.628	0.003–2348.8	0.780
		WMA	1.141	0.0002–5641.4	0.976
		MR-Egger	0.068	6.93E-10–6,636,867	0.786
Vitamin B12	7	IVW	0.936	0.819–1.070	0.333
		WMA	0.869	0.729–1.035	0.115
		MR-Egger	0.869	0.689–1.096	0.290
Vitamin D	94	IVW	1.071	0.939–1.219	0.304
		WMA	1.206	0.98–1.485	0.077
		MR-Egger	1.088	0.89–1.329	0.412

*OR* odds ratio, *CI* confidence interval, *SNP* single nucleotide polymorphism, *IVW* inverse-variance weighted, *WMA* weighted median approach.

*The SNPs of magnesium, selenium, vitamin B12 and vitamin D were P < 5 × 10^–8^; the SNPs of iron, vitamin A, zinc and folate were P < 5 × 10^–6^; the SNPs of copper was P < 5 × 10^–4^.

### Sensitivity analysis

The IVW method, the key MR analysis, was not impacted by heterogeneity in any of the studies, according to Cochrane’s Q test (magnesium Q statistic = 6.387; P = 0.270; iron Q statistic = 9.506; P = 0.392; copper Q statistic = 128.64; P = 0.467; zinc Q statistic = 2.843; P = 0.828; selenium Q statistic = 12.065; P = 0.281; folate Q statistic = 5.185; P = 0.637; vitamin A Q statistic = 6.604; P = 0.360; B12 Q statistic = 5.582; P = 0.472; and D Q statistic = 104.858; P = 0.169)(Table S2). Given that it was centered at zero for all MR analyses, the MR-Egger intercept did not demonstrate imbalanced horizontal pleiotropy (Pintercept > 0.05; Table S2). However, MR-PRESSO identified outlier SNPs for vitamin D (rs62007299, rs7652808) and B12 (rs1801222, rs602662) which our funnel plot and leave-one-out plot serve to illustrate (Table S2, Fig. S2).

## Discussion

In this research, we systematically assessed the causal relationships between nine micronutrients (magnesium, zinc, copper, iron, selenium, vitamins A, vitamins B12, vitamins D, and folate) and ADHD using a two-sample MR method. The findings revealed that no genetic evidence was found for a significant causal link between circulating concentrations of the remaining micronutrients and the risk of ADHD, which held up well in various sensitivity analyses. This may imply that reported epidemiological associations may be the result of confounding factors that cannot be controlled for.

Our results are contrary to those of a meta-analysis by Khoshbakht et al. who suggested a strong connection between vitamin D levels and the development of ADHD^40^. In another randomized controlled trial of micronutrient treatment for ADHD, Rucklidge et al. reported that ADHD patients with micronutrient treatment showed significant improvement in symptoms such as inattention, compared to the control group^41^. In addition, an observational study of 810 children by Huss et al. showed that magnesium and zinc intake had a positive effect on symptom improvement in children with ADHD, which is similar to those discovered in a research by Rucklidge et al.^42^. However, in our MR research, no significant association was found between circulating micronutrients and the occurrence of ADHD.

This inconsistency may be due to the following reasons: First, previous studies were mostly from observational studies and randomized controlled trials, and the results may have been influenced by confounding factors and reverse causation. In addition, previous studies still have the problem of small study size and sample size. Studies have shown that some trace elements, as key regulatory factors of serotonin synthesis, can regulate the synthesis of serotonin in a tissue-specific way, thus having a direct impact on the occurrence of ADHD and other mental diseases. According to Gall and Pertile et al.^43,44^, there is a connection between vitamin D and cognitive function. In the central nervous system, VDR is a nuclear receptor. 1,25(OH)_2_D3 binds to VDR to control the expression of dopaminergic genes. These findings confirm the hypothesis that the risk of ADHD might be mediated by circulating concentrations of micronutrients. However, these findings do not mean that changes in these micronutrient concentrations directly cause ADHD, and cannot resolve the previous controversy over whether micronutrient deficiencies are a cause or a result of ADHD. In summary, these findings support an association between ADHD and micronutrients, but, based on our MR study, there may not be a causal relationship between the two.

The advantages of our study are that, first of all, our data comes from largest available GWAS datasets, which has strong statistical power. Comparing MR to observational research, confounding factor bias can be reduced. Secondly, in order to reduce the bias caused by horizontal pleiotropy, this study used MR-PRESSO to detect broad horizontal pleiotropy in all results and eliminate abnormal SNPs. In addition, we used the Cochrane’s Q, MR-Egger test and leave-one-out methods to carry out sensitivity analysis, which guaranteed the reliability of the analysis results. And finally, in our study, the bias for confusion due to ancestry is reduced because our research was limited to participants of white European descent.

Our study has the following limitations. Firstly, while using a variety of MR methods to minimize the impact of pleiotropy on the data, we were unable to totally rule out the bias caused by unidentified pleiotropy. Second, due to the enlargement of the genome-wide significance level of certain phenotypes (copper, iron, zinc, folate, and vitamin A), this may affect how well we understand the results. Third, due to the minimal variance of the exposures explained by the SNP instruments or the short GWAS sample sizes, some of our MR analyses lacked the sensitivity to detect minor effects.

## Conclusions

In order to investigate the possible correlations between genetically predicted amounts of nine micronutrients and the risk of ADHD, we carried out the first comprehensive two-sample MR study. Our study found no genetic evidence of a significant causal relationship between circulating micronutrient concentrations and the risk of ADHD, and only suggested that there may be some association between copper levels and ADHD, but the significance of the findings was weak, so it is recommended that copper levels be used as a long-term monitoring indicator for further study. No heterogeneity or pleiotropy was found in sensitivity analysis. In order to reduce the incidence of ADHD and improve the quality of life of people with ADHD, more research can be focused on the potential link between micronutrients and ADHD in the future.

### Supplementary Information

Supplementary Figure S1.

Supplementary Figure S2.

Supplementary Table S1.

Supplementary Table S2.

## Data Availability

Data on ADHD was provided by the PGC Consortium investigators and can be downloade-d from https://www.med.unc.edu/pgc/. The summary statistics including iron (GWAS ID: ieu-a-1049), folate (GWAS ID: ukb-b-11349), and Vitamin A (GWAS ID: ukb-a-458) are publicly available from IEU OpenGWAS (https://gwas.mrcieu.ac.uk/). The GWAS summary-level data for magnesium (10.1371/journal.pgen.1001045), copper (10.1093/hmg/ddt239), selenium (10.1093/hmg/ddu546), zinc (10.1093/hmg/ddt239), Vitamin D (10.1038/s41467-020-15421-7), and Vitamin B12 (10.1002/ijc.32033) are publicly available from PubMed (https://pubmed.ncbi.nlm.nih.gov). The comprehensive details for GWAS database used in this research was presented in Supplementary Table S1.

## Abbreviations

ADHD: Attention deficit hyperactivity disorder
MR: Mendelian randomization
GWAS: Genome-wide association studies
SNPs: Single nucleotide polymorphisms
IVW: Inverse-variance weighted
OR: Odds ratio
CIs: Confidence intervals
SD: Standard deviation
IVs: Instrumental variables
PGC: Psychiatric Genomics Consortium
