# Supplementary material for: Appraising the role of circulating concentrations of micronutrients in attention deficit hyperactivity disorder: a Mendelian randomization study
Source: Sci Rep. 2023 Dec 9;13:21850. doi: 10.1038/s41598-023-49283-y (PMC10710398; doi:10.1038/s41598-023-49283-y)
Supplement: Supplementary file 2 — Supplementary Figure S2. [file 41598_2023_49283_MOESM2_ESM.pdf]

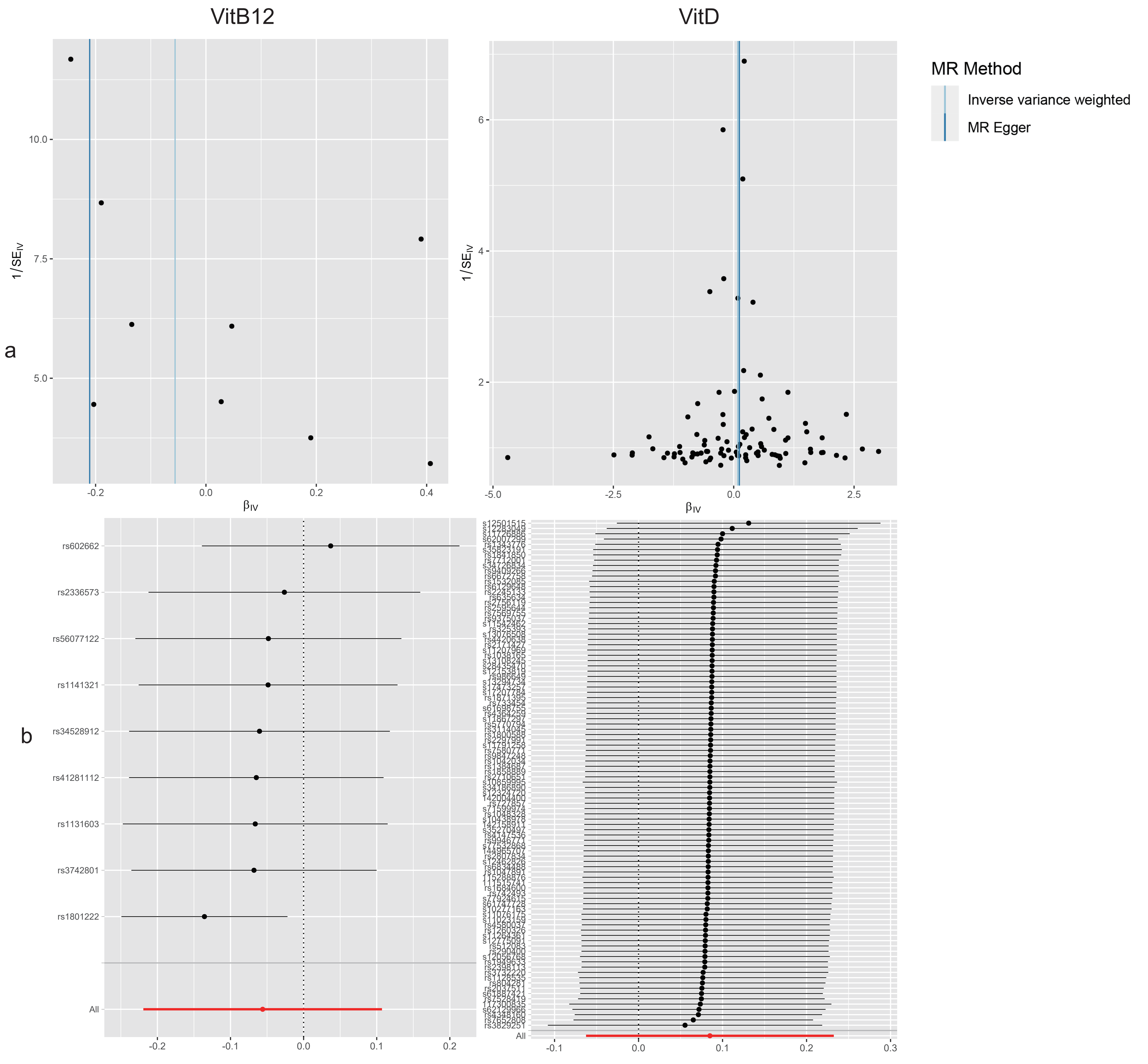

Figure S2 The Mendelian randomization study design.
ADHD, attention-deficit/hyperactivity disorder; MR, Mendelian Randomization; IVW, inverse-variance weighted.
