# Supplementary material for: Appraising the role of circulating concentrations of micronutrients in attention deficit hyperactivity disorder: a Mendelian randomization study
Source: Sci Rep. 2023 Dec 9;13:21850. doi: 10.1038/s41598-023-49283-y (PMC10710398; doi:10.1038/s41598-023-49283-y)
Supplement: Supplementary file 3 — Supplementary Table S1. [file 41598_2023_49283_MOESM3_ESM.docx]

**Table S1** Vitamins and minerals-associated SNPs used as genetic instruments in the Mendelian randomization analyses.

| **SNPs** | **Chr** | **Pos** | **Effect allele** | **Other allele** | **Beta** | **SE** | **P** | **R^2^** | **F** |
| --- | --- | --- | --- | --- | --- | --- | --- | --- | --- |
| **Minerals** |  |  |  |  |  |  |  |  |  |
| **Magnesium(6)** |  |  |  |  |  |  |  |  |  |
| rs11144134 | 9 | 74884880 | C | T | 0.011 | 0.001 | 8.00E-15 | 0.953 | 121 |
| rs13146355 | 4 | 76490987 | A | G | 0.005 | 0.001 | 6.00E-13 | 0.806 | 25 |
| rs3925584 | 11 | 30738788 | T | C | 0.006 | 0.001 | 5.20E-16 | 0.857 | 36 |
| rs4072037 | 1 | 155192276 | T | C | 0.010 | 0.001 | 2.00E-36 | 0.943 | 100 |
| rs448378 | 3 | 169383111 | A | G | 0.004 | 0.001 | 1.00E-08 | 0.727 | 16 |
| rs7965584 | 12 | 89912002 | A | G | 0.007 | 0.001 | 1.00E-16 | 0.891 | 49 |
| **Iron(10)** |  |  |  |  |  |  |  |  |  |
| rs1033478 | 6 | 56088216 | C | T | -0.055 | 0.011 | 3.63E-07 | 0.722 | 26 |
| rs12731 | 2 | 239087912 | A | G | 0.047 | 0.010 | 2.52E-06 | 0.691 | 22 |
| rs13038647 | 20 | 48974248 | C | T | 0.046 | 0.010 | 3.80E-06 | 0.682 | 21 |
| rs1525892 | 3 | 133484712 | A | G | 0.074 | 0.010 | 1.65E-12 | 0.834 | 50 |
| rs1800562 | 6 | 26093141 | A | G | 0.372 | 0.020 | 3.96E-77 | 0.972 | 347 |
| rs2075672 | 7 | 100240296 | G | A | 0.056 | 0.010 | 5.95E-08 | 0.745 | 29 |
| rs604302 | 8 | 37004569 | C | T | -0.058 | 0.012 | 3.07E-06 | 0.684 | 22 |
| rs6920211 | 6 | 135431318 | C | T | 0.054 | 0.012 | 3.14E-06 | 0.686 | 22 |
| rs7172337 | 15 | 61767743 | C | T | 0.052 | 0.011 | 2.63E-06 | 0.689 | 22 |
| rs855791 | 22 | 37462936 | G | A | 0.187 | 0.010 | 4.31E-77 | 0.972 | 342 |
| **Copper(129)** |  |  |  |  |  |  |  |  |  |
| rs10014072 | 4 | 113948790 | G | A | -0.164 | 0.034 | 1.13E-06 | 0.153 | 23 |
| rs1008488 | 6 | 9282739 | T | G | 0.416 | 0.114 | 2.59E-04 | 0.094 | 13 |
| rs10192667 | 2 | 15284746 | T | C | -0.104 | 0.028 | 1.93E-04 | 0.097 | 14 |
| rs10507003 | 12 | 92276779 | A | G | 0.225 | 0.063 | 3.77E-04 | 0.090 | 13 |
| rs10510837 | 3 | 60289842 | A | G | -0.167 | 0.041 | 5.24E-05 | 0.114 | 17 |
| rs10737805 | 1 | 247568051 | G | A | -0.121 | 0.034 | 3.77E-04 | 0.089 | 13 |
| rs10863512 | 1 | 220075215 | T | C | -0.393 | 0.106 | 2.10E-04 | 0.096 | 14 |
| rs10881505 | 1 | 108665931 | T | G | -0.126 | 0.029 | 1.75E-05 | 0.128 | 19 |
| rs10891215 | 11 | 110893949 | G | A | 0.115 | 0.03 | 1.22E-04 | 0.102 | 15 |
| rs10928190 | 2 | 144376033 | C | T | 0.093 | 0.027 | 4.64E-04 | 0.084 | 12 |
| rs10947096 | 6 | 30765895 | A | G | 0.146 | 0.042 | 4.62E-04 | 0.086 | 12 |
| rs10992047 | 9 | 94448572 | G | A | 0.100 | 0.027 | 1.77E-04 | 0.096 | 14 |
| rs11193249 | 10 | 108965797 | T | C | 0.187 | 0.051 | 2.89E-04 | 0.094 | 13 |
| rs1175550 | 1 | 3691528 | G | A | 0.198 | 0.032 | 5.03E-10 | 0.229 | 38 |
| rs11778954 | 8 | 65090848 | A | G | -0.131 | 0.032 | 3.66E-05 | 0.115 | 17 |
| rs11877452 | 18 | 36210636 | A | G | -0.108 | 0.028 | 9.76E-05 | 0.103 | 15 |
| rs12153606 | 5 | 84583769 | T | G | -0.159 | 0.034 | 2.50E-06 | 0.145 | 22 |
| rs12171510 | 5 | 91091363 | G | A | 0.852 | 0.242 | 4.34E-04 | 0.088 | 12 |
| rs12187771 | 5 | 89994983 | G | A | -0.138 | 0.038 | 2.92E-04 | 0.093 | 13 |
| rs12264890 | 10 | 5306085 | T | C | 0.310 | 0.086 | 3.36E-04 | 0.092 | 13 |
| rs12323487 | 14 | 28837771 | A | C | 0.299 | 0.07 | 1.74E-05 | 0.124 | 18 |
| rs12363407 | 11 | 45782272 | A | G | 0.144 | 0.039 | 2.56E-04 | 0.096 | 14 |
| rs12402211 | 1 | 164365427 | T | C | -0.170 | 0.049 | 4.81E-04 | 0.085 | 12 |
| rs12408827 | 1 | 75823198 | T | C | 0.183 | 0.052 | 4.33E-04 | 0.088 | 12 |
| rs12498514 | 4 | 166802117 | C | T | -0.113 | 0.028 | 7.24E-05 | 0.112 | 16 |
| rs12524956 | 6 | 40810472 | T | G | -0.268 | 0.071 | 1.58E-04 | 0.099 | 14 |
| rs12572984 | 10 | 67451570 | T | C | 0.212 | 0.058 | 2.54E-04 | 0.094 | 13 |
| rs12727765 | 1 | 242378894 | A | G | 0.112 | 0.031 | 2.50E-04 | 0.092 | 13 |
| rs12821339 | 12 | 127849576 | G | T | -0.288 | 0.076 | 1.37E-04 | 0.100 | 14 |
| rs12858977 | 13 | 79112171 | G | A | -0.284 | 0.078 | 2.91E-04 | 0.093 | 13 |
| rs12892771 | 14 | 85161541 | C | T | 1.044 | 0.283 | 2.21E-04 | 0.095 | 14 |
| rs12949459 | 17 | 35901374 | C | T | -0.135 | 0.034 | 5.88E-05 | 0.109 | 16 |
| rs13031525 | 2 | 155581862 | T | C | -0.233 | 0.064 | 2.89E-04 | 0.093 | 13 |
| rs13066500 | 3 | 22021394 | C | A | -0.106 | 0.027 | 9.72E-05 | 0.107 | 15 |
| rs13074172 | 3 | 142352978 | G | A | 0.121 | 0.027 | 7.16E-06 | 0.135 | 20 |
| rs13104623 | 4 | 55842065 | A | G | -0.120 | 0.028 | 1.58E-05 | 0.125 | 18 |
| rs13192569 | 6 | 123127597 | A | G | -0.156 | 0.041 | 1.35E-04 | 0.101 | 14 |
| rs13379919 | 15 | 69075034 | A | C | 0.302 | 0.086 | 4.67E-04 | 0.087 | 12 |
| rs1374459 | 2 | 23316591 | A | G | -0.107 | 0.027 | 5.87E-05 | 0.109 | 16 |
| rs1451538 | 15 | 85464630 | C | T | 0.305 | 0.086 | 4.05E-04 | 0.089 | 13 |
| rs1472949 | 4 | 123949556 | A | G | -0.230 | 0.065 | 4.15E-04 | 0.088 | 13 |
| rs1525817 | 7 | 46047726 | T | C | -0.227 | 0.065 | 4.94E-04 | 0.086 | 12 |
| rs1562183 | 8 | 34230497 | G | A | 0.358 | 0.1 | 3.56E-04 | 0.090 | 13 |
| rs1600909 | 5 | 113280580 | T | C | -0.201 | 0.053 | 1.51E-04 | 0.100 | 14 |
| rs16851939 | 1 | 203588311 | G | A | 0.230 | 0.06 | 1.30E-04 | 0.102 | 15 |
| rs16881257 | 4 | 28958838 | C | A | 0.216 | 0.06 | 3.13E-04 | 0.091 | 13 |
| rs16928808 | 9 | 9041784 | A | G | 0.103 | 0.029 | 4.46E-04 | 0.089 | 13 |
| rs16987935 | 20 | 41853763 | A | G | 1.164 | 0.293 | 7.33E-05 | 0.109 | 16 |
| rs17081569 | 18 | 67417576 | G | A | -0.183 | 0.051 | 2.95E-04 | 0.091 | 13 |
| rs17082912 | 13 | 26538585 | G | A | -0.203 | 0.056 | 2.98E-04 | 0.092 | 13 |
| rs17144483 | 7 | 21509745 | C | T | 0.305 | 0.078 | 9.78E-05 | 0.106 | 15 |
| rs17221996 | 16 | 12521923 | C | T | 0.176 | 0.043 | 4.17E-05 | 0.115 | 17 |
| rs17254216 | 14 | 56886829 | G | A | -0.134 | 0.035 | 1.46E-04 | 0.102 | 15 |
| rs17268037 | 3 | 98263849 | C | A | -0.131 | 0.037 | 3.77E-04 | 0.089 | 13 |
| rs17465447 | 5 | 165692655 | G | A | 0.125 | 0.035 | 3.71E-04 | 0.090 | 13 |
| rs1750098 | 13 | 101541480 | A | C | -0.096 | 0.028 | 4.81E-04 | 0.084 | 12 |
| rs17512311 | 1 | 73982357 | A | C | -0.195 | 0.051 | 1.32E-04 | 0.102 | 15 |
| rs17595345 | 19 | 30029436 | T | C | 0.285 | 0.073 | 9.81E-05 | 0.106 | 15 |
| rs17631367 | 8 | 17200201 | C | A | 0.171 | 0.04 | 2.13E-05 | 0.124 | 18 |
| rs17636424 | 11 | 100427659 | C | T | -0.198 | 0.053 | 1.72E-04 | 0.098 | 14 |
| rs17788636 | 7 | 93125921 | T | C | 0.132 | 0.033 | 6.34E-05 | 0.110 | 16 |
| rs17800852 | 12 | 1262829 | G | A | -0.222 | 0.056 | 6.57E-05 | 0.109 | 16 |
| rs1789981 | 8 | 107524282 | A | G | 0.126 | 0.034 | 2.00E-04 | 0.096 | 14 |
| rs183805 | 9 | 17029570 | G | T | 0.213 | 0.051 | 3.33E-05 | 0.119 | 17 |
| rs1861206 | 16 | 9751676 | T | C | -0.131 | 0.037 | 3.85E-04 | 0.089 | 13 |
| rs1867274 | 17 | 6456931 | T | C | 0.136 | 0.032 | 2.71E-05 | 0.123 | 18 |
| rs187445 | 16 | 84584323 | T | C | -0.117 | 0.03 | 1.03E-04 | 0.105 | 15 |
| rs1912136 | 11 | 24616743 | C | T | 0.113 | 0.029 | 1.03E-04 | 0.105 | 15 |
| rs2002250 | 2 | 86447484 | T | C | 0.098 | 0.028 | 4.58E-04 | 0.087 | 12 |
| rs2008341 | 7 | 157408733 | T | G | -0.100 | 0.027 | 2.25E-04 | 0.096 | 14 |
| rs2012451 | 22 | 31844373 | C | A | 0.092 | 0.026 | 4.66E-04 | 0.088 | 13 |
| rs2013795 | 19 | 55137303 | T | C | -0.116 | 0.032 | 2.29E-04 | 0.092 | 13 |
| rs218265 | 4 | 55408999 | C | T | -0.204 | 0.046 | 1.05E-05 | 0.132 | 20 |
| rs222331 | 7 | 10249494 | G | A | 0.133 | 0.035 | 1.51E-04 | 0.101 | 14 |
| rs2242157 | 1 | 5160662 | T | G | 0.103 | 0.027 | 1.19E-04 | 0.101 | 15 |
| rs2249287 | 1 | 237937874 | T | C | -0.159 | 0.039 | 4.21E-05 | 0.114 | 17 |
| rs2574951 | 10 | 52043987 | C | T | -0.294 | 0.077 | 1.33E-04 | 0.102 | 15 |
| rs2632839 | 8 | 18674701 | T | C | 0.157 | 0.04 | 8.96E-05 | 0.107 | 15 |
| rs2686336 | 12 | 24489008 | G | T | -0.155 | 0.041 | 1.75E-04 | 0.100 | 14 |
| rs2769264 | 1 | 151344741 | G | T | 0.313 | 0.034 | 2.63E-20 | 0.396 | 85 |
| rs2833477 | 21 | 33048864 | C | T | 0.170 | 0.042 | 5.79E-05 | 0.113 | 16 |
| rs3020343 | 6 | 152054363 | T | C | 0.114 | 0.028 | 5.06E-05 | 0.114 | 17 |
| rs3773971 | 3 | 190292028 | A | G | -0.129 | 0.037 | 4.24E-04 | 0.086 | 12 |
| rs3811444 | 1 | 248039451 | T | C | 0.134 | 0.03 | 1.12E-05 | 0.134 | 20 |
| rs3857536 | 6 | 66929048 | T | C | -0.129 | 0.028 | 4.08E-06 | 0.141 | 21 |
| rs4290555 | 18 | 5617122 | G | A | -0.118 | 0.033 | 3.72E-04 | 0.090 | 13 |
| rs4402988 | 4 | 145088692 | G | A | 0.149 | 0.04 | 2.13E-04 | 0.097 | 14 |
| rs441188 | 10 | 55296367 | T | C | -0.102 | 0.028 | 2.58E-04 | 0.093 | 13 |
| rs4817026 | 21 | 26917355 | T | C | 0.240 | 0.064 | 1.89E-04 | 0.098 | 14 |
| rs4910317 | 11 | 11337655 | G | A | -0.200 | 0.047 | 2.20E-05 | 0.123 | 18 |
| rs515458 | 11 | 70490207 | G | A | 0.113 | 0.032 | 3.42E-04 | 0.088 | 12 |
| rs517176 | 1 | 37295408 | C | T | 0.131 | 0.037 | 4.42E-04 | 0.089 | 13 |
| rs567931 | 9 | 101133518 | T | C | 0.117 | 0.03 | 9.17E-05 | 0.105 | 15 |
| rs572585 | 2 | 119354478 | C | T | -0.137 | 0.031 | 9.13E-06 | 0.131 | 20 |
| rs5769396 | 22 | 49473220 | A | C | 0.194 | 0.055 | 4.14E-04 | 0.088 | 12 |
| rs6006800 | 22 | 46133853 | G | A | -0.123 | 0.035 | 4.17E-04 | 0.087 | 12 |
| rs6066646 | 20 | 46778604 | A | C | -0.094 | 0.027 | 4.08E-04 | 0.086 | 12 |
| rs6089571 | 20 | 60534693 | G | A | 0.119 | 0.029 | 4.82E-05 | 0.115 | 17 |
| rs628462 | 1 | 10511544 | T | C | 0.105 | 0.028 | 2.13E-04 | 0.098 | 14 |
| rs6440616 | 3 | 149197860 | T | G | 0.408 | 0.115 | 4.02E-04 | 0.089 | 13 |
| rs6480814 | 10 | 78165245 | C | T | 0.452 | 0.109 | 3.52E-05 | 0.118 | 17 |
| rs6553249 | 4 | 189828337 | T | C | 0.142 | 0.038 | 1.92E-04 | 0.098 | 14 |
| rs6693567 | 1 | 150510660 | T | C | 0.115 | 0.032 | 4.24E-04 | 0.091 | 13 |
| rs6841508 | 4 | 180313511 | A | G | 0.149 | 0.037 | 6.92E-05 | 0.112 | 16 |
| rs7099752 | 10 | 129751331 | A | G | -0.195 | 0.05 | 1.09E-04 | 0.105 | 15 |
| rs710003 | 14 | 59239300 | G | A | -0.156 | 0.04 | 9.56E-05 | 0.105 | 15 |
| rs714191 | 22 | 29145411 | C | T | 0.126 | 0.036 | 4.62E-04 | 0.087 | 12 |
| rs7206796 | 16 | 56381277 | T | C | -0.152 | 0.034 | 9.26E-06 | 0.134 | 20 |
| rs7226121 | 17 | 28208125 | A | C | 0.103 | 0.027 | 1.20E-04 | 0.101 | 15 |
| rs7475679 | 10 | 19343732 | G | A | -0.114 | 0.032 | 3.35E-04 | 0.090 | 13 |
| rs7608158 | 2 | 222146655 | A | G | -0.209 | 0.056 | 1.67E-04 | 0.097 | 14 |
| rs7632830 | 3 | 29764777 | A | G | -0.158 | 0.041 | 1.35E-04 | 0.103 | 15 |
| rs764560 | 7 | 27359068 | T | C | -0.128 | 0.028 | 6.42E-06 | 0.139 | 21 |
| rs7654880 | 4 | 4411166 | C | T | -0.154 | 0.042 | 2.35E-04 | 0.094 | 13 |
| rs7675330 | 4 | 15963113 | A | G | 0.102 | 0.027 | 1.25E-04 | 0.100 | 14 |
| rs7814505 | 8 | 24536186 | G | A | -0.144 | 0.036 | 7.41E-05 | 0.110 | 16 |
| rs8138794 | 22 | 43866756 | A | G | 0.587 | 0.156 | 1.63E-04 | 0.099 | 14 |
| rs871772 | 15 | 82379182 | A | G | -0.229 | 0.058 | 7.67E-05 | 0.108 | 16 |
| rs923623 | 5 | 29006269 | A | G | -0.101 | 0.028 | 3.34E-04 | 0.092 | 13 |
| rs9291932 | 5 | 68043243 | C | T | -0.128 | 0.031 | 2.91E-05 | 0.117 | 17 |
| rs9324493 | 8 | 139727981 | G | A | -0.177 | 0.039 | 5.94E-06 | 0.138 | 21 |
| rs9409226 | 9 | 122850776 | A | C | -0.221 | 0.058 | 1.44E-04 | 0.101 | 15 |
| rs9474391 | 6 | 52982348 | A | C | -0.455 | 0.109 | 2.78E-05 | 0.119 | 17 |
| rs9530372 | 13 | 75294209 | C | A | -0.113 | 0.032 | 3.47E-04 | 0.088 | 12 |
| rs9577550 | 13 | 114096891 | C | T | 0.143 | 0.041 | 4.53E-04 | 0.086 | 12 |
| rs9587495 | 13 | 108702721 | G | A | 0.123 | 0.033 | 1.98E-04 | 0.097 | 14 |
| rs9808529 | 2 | 229968218 | C | T | 0.101 | 0.027 | 1.70E-04 | 0.098 | 14 |
| rs9879624 | 3 | 1306926 | A | G | 0.150 | 0.042 | 2.96E-04 | 0.090 | 13 |
| rs9899091 | 17 | 78299331 | C | T | -0.404 | 0.095 | 2.12E-05 | 0.123 | 18 |
| **Zinc(7)** |  |  |  |  |  |  |  |  |  |
| rs10484100 | 14 | 86817096 | G | A | -0.209 | 0.045 | 3.30E-06 | 0.755 | 22 |
| rs11232535 | 11 | 80928809 | C | T | 0.325 | 0.065 | 6.73E-07 | 0.781 | 25 |
| rs11763353 | 7 | 15630871 | G | A | -0.192 | 0.039 | 6.90E-07 | 0.776 | 24 |
| rs1532423 | 8 | 86268313 | G | A | -0.178 | 0.026 | 6.40E-12 | 0.870 | 47 |
| rs2120019 | 15 | 75334184 | C | T | -0.287 | 0.033 | 1.55E-18 | 0.915 | 76 |
| rs4333127 | 4 | 5930033 | A | G | 0.218 | 0.047 | 3.00E-06 | 0.755 | 22 |
| rs7148590 | 14 | 65473196 | A | G | -0.140 | 0.026 | 1.37E-07 | 0.806 | 29 |
| **Selenium(11)** |  |  |  |  |  |  |  |  |  |
| rs10944 | 5 | 78385845 | T | G | 0.258 | 0.020 | 1.13E-36 | 0.936 | 160 |
| rs11951068 | 5 | 78304314 | A | G | 0.268 | 0.040 | 1.86E-11 | 0.804 | 45 |
| rs1789953 | 21 | 44482936 | T | C | 0.162 | 0.029 | 3.40E-08 | 0.735 | 30 |
| rs234709 | 21 | 44486964 | T | C | -0.120 | 0.020 | 5.23E-09 | 0.756 | 34 |
| rs3797535 | 5 | 78300397 | T | C | 0.298 | 0.038 | 2.05E-15 | 0.851 | 63 |
| rs567754 | 5 | 78416416 | T | C | -0.196 | 0.022 | 8.38E-20 | 0.883 | 83 |
| rs6586282 | 21 | 44478497 | T | C | -0.160 | 0.027 | 3.96E-09 | 0.759 | 35 |
| rs672413 | 5 | 78278229 | A | G | 0.164 | 0.022 | 5.21E-14 | 0.838 | 57 |
| rs6859667 | 5 | 78745042 | T | C | -0.360 | 0.052 | 4.40E-12 | 0.813 | 48 |
| rs705415 | 5 | 78291960 | T | C | -0.200 | 0.032 | 4.64E-10 | 0.779 | 39 |
| rs921943 | 5 | 78316476 | T | C | 0.295 | 0.022 | 9.00E-28 | 0.940 | 173 |
| **Folate(8)** |  |  |  |  |  |  |  |  |  |
| rs139588363 | 2 | 140739695 | C | T | 0.054 | 0.012 | 4.10E-06 | 0.726 | 21 |
| rs16956822 | 17 | 7499349 | A | G | -0.079 | 0.017 | 3.90E-06 | 0.727 | 21 |
| rs2449166 | 8 | 3463535 | T | C | 0.025 | 0.005 | 4.00E-06 | 0.727 | 21 |
| rs7074988 | 10 | 87848038 | G | A | -0.051 | 0.011 | 4.00E-06 | 0.727 | 21 |
| rs76630415 | 7 | 14144445 | G | T | -0.037 | 0.007 | 2.40E-08 | 0.795 | 31 |
| rs76802001 | 22 | 31013399 | A | G | -0.068 | 0.015 | 4.60E-06 | 0.724 | 21 |
| rs78074774 | 2 | 34515489 | T | C | 0.060 | 0.013 | 4.90E-06 | 0.723 | 21 |
| rs8085166 | 18 | 3016615 | G | A | 0.028 | 0.006 | 1.70E-06 | 0.741 | 23 |
| **Vitamins** |  |  |  |  |  |  |  |  |  |
| **Vitamin A(7)** |  |  |  |  |  |  |  |  |  |
| rs1849759 | 3 | 80438940 | A | G | 0.002 | 0.000 | 3.24E-06 | 0.756 | 22 |
| rs189776215 | 9 | 16693849 | A | G | 0.007 | 0.002 | 3.82E-06 | 0.753 | 21 |
| rs2378739 | 9 | 90009875 | A | C | 0.002 | 0.000 | 3.77E-06 | 0.753 | 21 |
| rs684396 | 11 | 63161012 | C | T | 0.005 | 0.001 | 2.96E-06 | 0.757 | 22 |
| rs72833036 | 6 | 24351057 | G | A | 0.004 | 0.001 | 2.15E-08 | 0.817 | 31 |
| rs77606808 | 1 | 163584207 | C | T | 0.003 | 0.001 | 4.56E-06 | 0.750 | 21 |
| rs8132245 | 21 | 34516359 | T | G | 0.002 | 0.000 | 8.01E-07 | 0.777 | 24 |
| **Vitamin B12(7)** |  |  |  |  |  |  |  |  |  |
| rs1131603 | 22 | 31018975 | C | T | 0.190 | 0.017 | 4.30E-28 | 0.947 | 125 |
| rs1141321 | 6 | 49412433 | C | T | 0.061 | 0.007 | 1.40E-16 | 0.916 | 76 |
| rs2336573 | 19 | 8367709 | T | C | 0.320 | 0.021 | 1.10E-51 | 0.971 | 232 |
| rs34528912 | 11 | 59631535 | T | C | 0.170 | 0.021 | 2.10E-15 | 0.903 | 66 |
| rs3742801 | 14 | 74759006 | T | C | 0.045 | 0.008 | 5.30E-08 | 0.819 | 32 |
| rs41281112 | 13 | 100518634 | C | T | 0.170 | 0.016 | 9.60E-27 | 0.942 | 113 |
| rs56077122 | 10 | 17207015 | A | C | 0.087 | 0.009 | 4.80E-21 | 0.930 | 93 |
| **Vitamin D(94)** |  |  |  |  |  |  |  |  |  |
| rs10277163 | 7 | 21569089 | G | A | -0.014 | 0.002 | 1.08E-09 | 0.283 | 37 |
| rs1038165 | 12 | 68665940 | T | C | 0.012 | 0.002 | 2.15E-08 | 0.250 | 31 |
| rs1042034 | 2 | 21225281 | T | C | -0.015 | 0.003 | 1.45E-09 | 0.280 | 37 |
| rs10438978 | 18 | 47158186 | C | T | -0.017 | 0.003 | 7.34E-11 | 0.311 | 42 |
| rs1047891 | 2 | 211540507 | A | C | -0.013 | 0.002 | 7.96E-10 | 0.287 | 38 |
| rs1048328 | 19 | 51527364 | A | G | 0.031 | 0.004 | 5.58E-17 | 0.427 | 70 |
| rs10859995 | 12 | 96375682 | C | T | -0.044 | 0.002 | 4.60E-100 | 0.827 | 451 |
| rs11023159 | 11 | 14262063 | C | T | 0.048 | 0.006 | 3.73E-17 | 0.430 | 71 |
| rs11076175 | 16 | 57006378 | G | A | 0.023 | 0.003 | 9.64E-18 | 0.439 | 74 |
| rs111515741 | 11 | 14370944 | A | G | -0.049 | 0.008 | 3.95E-10 | 0.294 | 39 |
| rs11207969 | 1 | 62911751 | G | A | 0.021 | 0.002 | 7.14E-23 | 0.508 | 97 |
| rs11264361 | 1 | 155289545 | G | T | 0.017 | 0.002 | 7.97E-14 | 0.373 | 56 |
| rs1128535 | 3 | 49866392 | T | C | 0.016 | 0.002 | 6.06E-16 | 0.410 | 65 |
| rs115288876 | 1 | 152000117 | A | G | 0.079 | 0.005 | 2.36E-56 | 0.727 | 250 |
| rs11542462 | 16 | 82033810 | A | G | -0.025 | 0.003 | 9.72E-17 | 0.423 | 69 |
| rs11726886 | 4 | 72822599 | A | C | -0.054 | 0.002 | 3.08E-125 | 0.858 | 567 |
| rs117300835 | 11 | 15118975 | A | G | -0.335 | 0.009 | 1.00E-200 | 0.938 | 1429 |
| rs11791258 | 9 | 107632644 | A | G | 0.014 | 0.003 | 4.85E-08 | 0.241 | 30 |
| rs11867297 | 17 | 66433493 | T | C | 0.014 | 0.002 | 1.01E-10 | 0.308 | 42 |
| rs12056768 | 8 | 116988527 | G | T | -0.023 | 0.002 | 2.65E-29 | 0.573 | 126 |
| rs12153819 | 6 | 83773049 | T | C | -0.018 | 0.003 | 8.16E-09 | 0.261 | 33 |
| rs12283049 | 11 | 14690192 | G | A | -0.056 | 0.002 | 9.62E-122 | 0.854 | 551 |
| rs12324720 | 15 | 64092140 | A | G | -0.015 | 0.003 | 2.45E-08 | 0.249 | 31 |
| rs12462826 | 19 | 11955767 | A | G | -0.013 | 0.002 | 4.18E-10 | 0.293 | 39 |
| rs12501515 | 4 | 72592838 | A | G | -0.079 | 0.002 | 1.00E-200 | 0.939 | 1456 |
| rs1260326 | 2 | 27730940 | C | T | 0.020 | 0.002 | 1.96E-21 | 0.490 | 90 |
| rs12775091 | 10 | 91524012 | T | C | 0.016 | 0.002 | 3.33E-10 | 0.296 | 39 |
| rs13076508 | 3 | 52407805 | C | T | 0.025 | 0.005 | 2.78E-08 | 0.247 | 31 |
| rs13108245 | 4 | 57790205 | G | A | -0.012 | 0.002 | 4.63E-09 | 0.268 | 34 |
| rs13294734 | 9 | 80710910 | T | C | 0.013 | 0.002 | 1.02E-09 | 0.284 | 37 |
| rs1343776 | 1 | 41757718 | A | G | 0.018 | 0.002 | 1.62E-13 | 0.367 | 54 |
| rs1384687 | 8 | 61525963 | A | G | -0.017 | 0.003 | 1.82E-08 | 0.252 | 32 |
| rs142004400 | 14 | 50829560 | C | A | -0.031 | 0.006 | 3.01E-08 | 0.246 | 31 |
| rs142158911 | 19 | 11190534 | A | G | 0.026 | 0.003 | 4.43E-16 | 0.413 | 66 |
| rs144965707 | 11 | 14059511 | A | G | -0.035 | 0.004 | 1.52E-16 | 0.420 | 68 |
| rs1532085 | 15 | 58683366 | G | A | 0.025 | 0.002 | 8.60E-34 | 0.610 | 147 |
| rs1684600 | 16 | 4594671 | T | C | -0.013 | 0.002 | 1.59E-08 | 0.254 | 32 |
| rs17207784 | 6 | 22768668 | C | T | -0.013 | 0.002 | 5.14E-10 | 0.291 | 39 |
| rs17473257 | 11 | 14283186 | A | G | -0.061 | 0.008 | 4.59E-15 | 0.395 | 61 |
| rs1800588 | 15 | 58723675 | T | C | -0.031 | 0.002 | 4.73E-35 | 0.619 | 153 |
| rs1841850 | 20 | 52718179 | C | A | 0.030 | 0.003 | 6.73E-22 | 0.496 | 93 |
| rs1858889 | 7 | 107117447 | C | A | 0.013 | 0.002 | 3.49E-11 | 0.318 | 44 |
| rs1871395 | 12 | 21352315 | G | A | -0.020 | 0.003 | 5.72E-13 | 0.356 | 52 |
| rs1949633 | 3 | 153758806 | C | T | 0.011 | 0.002 | 4.45E-08 | 0.242 | 30 |
| rs2037511 | 18 | 61366207 | A | G | 0.018 | 0.003 | 9.41E-11 | 0.309 | 42 |
| rs2171427 | 12 | 24822154 | A | G | -0.017 | 0.003 | 4.26E-09 | 0.268 | 35 |
| rs2245133 | 6 | 131931092 | C | T | -0.021 | 0.003 | 7.80E-15 | 0.391 | 60 |
| rs2297991 | 10 | 113913222 | C | T | 0.013 | 0.002 | 1.57E-08 | 0.254 | 32 |
| rs2398113 | 10 | 10076429 | G | A | -0.012 | 0.002 | 1.10E-08 | 0.258 | 33 |
| rs2595644 | 7 | 43980540 | T | G | -0.012 | 0.002 | 4.97E-09 | 0.267 | 34 |
| rs2710651 | 2 | 63166379 | A | G | -0.012 | 0.002 | 1.23E-08 | 0.257 | 32 |
| rs2756119 | 14 | 104001517 | A | G | 0.012 | 0.002 | 8.71E-09 | 0.260 | 33 |
| rs2807834 | 1 | 220970593 | G | T | -0.015 | 0.002 | 5.66E-12 | 0.335 | 47 |
| rs28435470 | 12 | 133067473 | A | G | -0.012 | 0.002 | 3.29E-08 | 0.245 | 31 |
| rs290400 | 20 | 52698179 | A | G | -0.013 | 0.002 | 1.41E-09 | 0.281 | 37 |
| rs3114045 | 4 | 100252560 | C | T | -0.022 | 0.003 | 1.00E-13 | 0.371 | 55 |
| rs325393 | 15 | 100229260 | T | G | -0.014 | 0.002 | 2.03E-09 | 0.277 | 36 |
| rs34186890 | 3 | 141720712 | G | A | -0.016 | 0.002 | 1.33E-11 | 0.327 | 46 |
| rs34726834 | 8 | 25889606 | T | C | 0.014 | 0.002 | 2.42E-09 | 0.275 | 36 |
| rs35270497 | 2 | 38259872 | T | C | 0.016 | 0.003 | 5.08E-09 | 0.267 | 34 |
| rs35823191 | 1 | 17560123 | C | T | -0.023 | 0.002 | 1.65E-27 | 0.557 | 118 |
| rs3732220 | 2 | 234627048 | A | G | -0.048 | 0.004 | 1.31E-39 | 0.649 | 173 |
| rs3829251 | 11 | 71194559 | A | G | -0.114 | 0.003 | 1.00E-200 | 0.940 | 1475 |
| rs4147536 | 4 | 100239112 | C | A | -0.015 | 0.002 | 2.76E-09 | 0.273 | 35 |
| rs4348160 | 4 | 70017531 | G | T | -0.026 | 0.002 | 6.62E-33 | 0.603 | 143 |
| rs4364259 | 4 | 15892159 | A | G | 0.017 | 0.003 | 1.86E-11 | 0.324 | 45 |
| rs4420638 | 19 | 45422946 | G | A | -0.019 | 0.003 | 3.95E-13 | 0.359 | 53 |
| rs4580037 | 13 | 55702646 | C | A | -0.014 | 0.002 | 1.68E-09 | 0.279 | 36 |
| rs512083 | 1 | 46027355 | C | T | 0.012 | 0.002 | 2.23E-09 | 0.276 | 36 |
| rs5770794 | 22 | 50880781 | T | C | -0.013 | 0.002 | 1.74E-09 | 0.278 | 36 |
| rs6129648 | 20 | 39231118 | G | A | 0.014 | 0.002 | 2.44E-11 | 0.322 | 45 |
| rs61698755 | 17 | 79257880 | C | T | -0.011 | 0.002 | 2.25E-08 | 0.250 | 31 |
| rs61747728 | 1 | 179526214 | T | C | 0.030 | 0.005 | 8.83E-09 | 0.260 | 33 |
| rs62129966 | 19 | 48374950 | A | C | 0.061 | 0.003 | 1.60E-108 | 0.839 | 490 |
| rs635634 | 9 | 136155000 | T | C | -0.015 | 0.003 | 7.55E-09 | 0.262 | 33 |
| rs6672758 | 1 | 230303512 | T | C | 0.016 | 0.003 | 2.04E-10 | 0.301 | 40 |
| rs6834488 | 4 | 88178919 | T | C | -0.014 | 0.002 | 2.26E-12 | 0.344 | 49 |
| rs71599974 | 4 | 71765339 | G | A | 0.026 | 0.003 | 2.39E-19 | 0.462 | 81 |
| rs727857 | 2 | 58981967 | A | G | -0.012 | 0.002 | 9.27E-09 | 0.260 | 33 |
| rs733454 | 11 | 76477721 | T | C | 0.019 | 0.003 | 2.93E-08 | 0.247 | 31 |
| rs742493 | 6 | 40998167 | C | T | 0.018 | 0.003 | 1.04E-08 | 0.258 | 33 |
| rs7528419 | 1 | 109817192 | G | A | 0.022 | 0.002 | 8.17E-19 | 0.455 | 78 |
| rs7569755 | 2 | 118648261 | A | G | 0.014 | 0.002 | 1.49E-09 | 0.280 | 37 |
| rs7580771 | 2 | 101428119 | T | G | -0.017 | 0.003 | 5.15E-10 | 0.291 | 39 |
| rs7712001 | 5 | 148020950 | G | T | 0.012 | 0.002 | 7.05E-09 | 0.263 | 34 |
| rs77532868 | 10 | 88081438 | T | C | 0.026 | 0.005 | 1.28E-08 | 0.256 | 32 |
| rs77924615 | 16 | 20392332 | A | G | -0.015 | 0.003 | 3.94E-09 | 0.269 | 35 |
| rs7955128 | 12 | 38684121 | T | A | 0.013 | 0.002 | 1.48E-10 | 0.304 | 41 |
| rs804281 | 8 | 11611865 | G | A | 0.016 | 0.002 | 1.20E-14 | 0.388 | 60 |
| rs9375037 | 6 | 121856794 | C | A | 0.012 | 0.002 | 1.21E-08 | 0.257 | 32 |
| rs9409266 | 9 | 125745042 | A | G | -0.017 | 0.003 | 1.24E-08 | 0.256 | 32 |
| rs9847248 | 3 | 18804655 | A | G | -0.012 | 0.002 | 4.19E-08 | 0.242 | 30 |
| rs986649 | 5 | 118668050 | G | A | 0.013 | 0.002 | 3.51E-09 | 0.271 | 35 |
| rs9946771 | 18 | 28918628 | T | C | -0.023 | 0.004 | 9.47E-09 | 0.260 | 33 |

* The SNPs of magnesium, selenium, vitamin B12 and vitamin D were P<5×10^-8^; the SNPs of iron, vitamin A, zinc and folate were P<5×10^-6^; the SNPs of copper was P<5×10^-4^.

SNPs, single nucleotide polymorphisms; Chr, chromosome; Pos, position; EAF, effect allele frequency; SE, standard error.
