# Supplementary material for: Appraising the role of circulating concentrations of micronutrients in attention deficit hyperactivity disorder: a Mendelian randomization study
Source: Sci Rep. 2023 Dec 9;13:21850. doi: 10.1038/s41598-023-49283-y (PMC10710398; doi:10.1038/s41598-023-49283-y)
Supplement: Supplementary file 4 — Supplementary Table S2. [file 41598_2023_49283_MOESM4_ESM.doc]

**Table S2** Cochran’s Q test and MR-Egger intercept Mendelian randomization analyses of micro-nutrients and ADHD.

|  | **Exposure** | **Snps** | **Heterogeneity** | | **Pleiotropy** | | | | **MR-PRESSO** | |
| --- | --- | --- | --- | --- | --- | --- | --- | --- | --- | --- |
|  |  |  | Cochran’s Q statistic^1^ | **P-value** | MR-Egger intercept^2^ | **SE** | **P-value** | Global Test^3^ | | **P-value** |
| **magnesium** | | 6 | 6.387 | 0.270 | -0.019 | 0.021 | 0.426 | 8.637 | | 0.376 |
|  | **iron** | 13 | 9.506 | 0.392 | -0.002 | 0.008 | 0.788 | 10.583 | | 0.557 |
|  | **copper** | 129 | 128.640 | 0.467 | 0.002 | 0.003 | 0.595 | 154.345 | | 0.495 |
|  | **zinc** | 7 | 2.843 | 0.828 | -0.014 | 0.025 | 0.604 | 4.801 | | 0.822 |
|  | **selenium** | 11 | 12.065 | 0.281 | 0.003 | 0.020 | 0.883 | 14.847 | | 0.292 |
|  | **folate** | 8 | 5.185 | 0.637 | -0.028 | 0.019 | 0.191 | 6.639 | | 0.681 |
|  | **Vit A** | 7 | 6.604 | 0.360 | 0.010 | 0.023 | 0.2689 | 12.345 | | 0.235 |
|  | **Vit B12** | 7 | 5.582 | 0.472 | 0.009 | 0.013 | 0.479 | 34.806 | | 0.007 |
|  | **Vit D** | 94 | 104.858 | 0.169 | -0.0006 | 0.003 | 0.836 | 154.166 | | 0.004 |

| | ^1^The Cochran’s Q test is a statistical test for heterogeneity. This statistic could not be calculated for Vitamin A,  because less than two SNPs were available. | | --- | |
| --- | --- |

^2^The intercept term from the MR-Egger regression method is a statistical test of horizontal pleiotropy. This statistic could not be calculated for Vitamin A, copper and zinc, because less than three SNPs were available.

| ^3^The MR-PRESSO method detected the existence of outlier IVs that may have horizontal pleiotropy  through the global test. This statistic could not be calculated for iron, copper, zinc, folate, Vitamin A,  because less than four SNPs were available. |
| --- |

* The SNPs of magnesium, selenium, vitamin B12 and vitamin D were P<5×10^-8^; the SNPs of iron, vitamin A, zinc and folate were P<5×10^-6^; the SNPs of copper was P<5×10^-4^.

SNPs, single nucleotide polymorphisms;SE, standard error.
